# Splenectomy Improves Outcome of Intracerebral Hemorrhage

**DOI:** 10.1096/fj.202500191R

**Published:** 2025-04-18

**Authors:** Chun Hin Leung, Hei Tung Shek, Jiaxin Liu, Karrie M. Kiang, Gilberto Ka‐Kit Leung

**Affiliations:** ^1^ Department of Surgery, School of Clinical Medicine, LKS Faculty of Medicine The University of Hong Kong Hong Kong Hong Kong; ^2^ The State Key Laboratory of Brain and Cognitive Sciences The University of Hong Kong Hong Kong Hong Kong

## Abstract

The acute splenic response triggers a surge of immune cells and pro‐inflammatory mediators, exacerbating secondary brain injury and worsening the outcome after intracerebral hemorrhage (ICH). Splenectomy confers benefits in experimental ischaemic stroke, but its effects on ICH are not known. We conducted a proof‐of‐concept study to test the hypothesis that a prior splenectomy would improve behavioral outcomes after ICH. Adult C57BL/6N mice with collagenase‐induced ICH that had undergone splenectomy two weeks prior were compared with those with intact spleens. Motor function, haematoma size, cerebral oedema, intracerebral and peripheral blood neutrophil counts were evaluated. Splenectomised mice had lower neutrophil counts in peripheral blood and peri‐haematoma regions of the brain. They performed better on the modified Neurological Severity Score during the first week post‐ICH and for a shorter duration on rotarod and cylinder tests. Haematomas in non‐splenectomised animals showed expansion after the initial hemorrhage and were larger than in splenectomised animals, with more severe surrounding oedema and greater mass effect. This is the first report on the beneficial effects of splenectomy in experimental ICH. Further studies on the optimal timing and mode of splenic response ablation are justified to assess its translational potential.

## Graphical abstract

In this in vivo study, mice were divided into splenectomy and laparotomy groups (control), and ICH was induced 2 weeks after. It is demonstrated that ICH products such as blood trigger an acute splenic response, leading to immune cell infiltration into the peri‐hematomal region, which in turn causes neuroinflammation and damage to blood‐brain barrier. This subsequently results in hematoma expansion and cerebral edema seen in brain sections and MRI, respectively, forming a vicious cycle of brain‐spleen interaction.

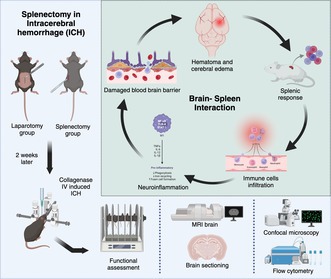

## 1 Introduction

The spleen, being a major source of peripheral immune cells, contributes to the systemic inflammatory response and secondary brain injury after intracerebral hemorrhage (ICH) [1]. While splenectomy has been shown to reduce infarct volume and neurological deficits in experimental ischaemic stroke [2, 3, 4], its effects on ICH have not been examined [5, 6, 7]. We conducted a proof‐of‐concept study to test the hypothesis that a prior splenectomy would reduce cerebral infiltration by neutrophils, reduce blood–brain barrier (BBB) disruption and cerebral oedema, and improve neurological outcome after ICH.

## 2 Methods

### 2.1 Animal Study

Twelve‐week‐old male C57BL/6N mice, weighing 21–31 g, were randomly divided into four groups using online tools. Experimental protocols were approved by the Committee on the Use of Live Animals in Teaching and Research of our institution. Blinding was not done due to obvious behavioral differences between the groups and a small number of investigators. At least 3 animals were assigned per group to ensure adequate study power.

### 2.2 ICH Induction

A right‐sided burr hole was created with a micromotor drill. 0.5 μL of 0.08 U/μL type IV bacterial collagenase in 0.9% normal saline was injected into the right striatum. The sham‐ICH group underwent the same procedure without collagenase injection.

### 2.3 Splenectomy

Spleens were removed 14 days before ICH induction. Through a midline laparotomy, the spleen was resected with vessel ligations. The sham‐surgery group underwent laparotomy without splenic resection.

### 2.4 Functional Assessment

The modified Neurological Severity Score (mNSS) was used to assess motor, reflex, and balance [8]. After pre‐test training, the rotarod test and cylinder test were performed post‐ICH. The rotarod gradually rotated from 4–40 rpm over 5 min [9]. A latency‐to‐fall period was computed as the time taken from the start of the rotation to its termination. The average of 3 runs was taken. The cylinder test assessed the asymmetry of spontaneous contact with the inner wall between forelimbs. The experiment ended when 10 min lapsed or 20 contacts were reached. Mice with fewer than 10 contacts were excluded.

### 2.5 Haematoma Size

ICH volume was calculated by multiplying the area of haematoma by the thickness in each slide (1 mm) using ImageJ software. Hemoglobin content was determined by mixing the supernatant of sonicated brain specimens with Drabkin's reagent and measuring the optical density of the solution with reference to a standard curve.

### 2.6 Magnetic Resonance Imaging (MRI)

Animals' brains were imaged with a 7‐Tesla MRI machine: repetition time (TR) = 4200 ms, time to echo = 12 ms, T2‐weighted image, 20 mm × 20 mm for field of view and 0.5 mm slice thickness.

### 2.7 Brain Water Content

Each extracted cerebral hemisphere was weighed immediately after extraction (wet weight), then re‐weighed after being dried for 3 days (dry weight).

### 2.8 Immunofluorescence Staining

Mice brains were harvested and cryosectioned at a thickness of 10 μm, incubated with anti‐CD11b rat monoclonal antibody, and stained with DAPI. Images were taken with an LSM780 confocal microscope.

### 2.9 Flow Cytometry

Single cell suspensions were made from peripheral blood and brain samples. Samples were first stained with Zombie Violet Fixable Viability Kit, then incubated with TruStain FcX anti‐mouse CD16/32 antibody. A cocktail of antibodies: CD11b, F4/80, and Ly6G was used to stain neutrophils.

### 2.10 Statistical Analyses

Data were shown as mean ± standard error of the mean (SEM). Statistical significance in comparing two groups was tested with two‐tailed unpaired Student's *t*‐test (with Welch's correction or Mann–Whitney modification). One‐way ANOVA or repeated measures ANOVA followed by Bonferroni's multiple comparisons test and Kruskal–Wallis test followed by post hoc Dunn's multiple comparisons test were used to compare the means of three or more groups for datasets with parametric and non‐parametric distributions, respectively. A *p*‐value < 0.05 is considered statistically significant. GraphPad Prism (version 9.3.1) software was used.

Graphical abstract is created using BioRender (http://biorender.com).

## 3 Results

**FIGURE 1:**
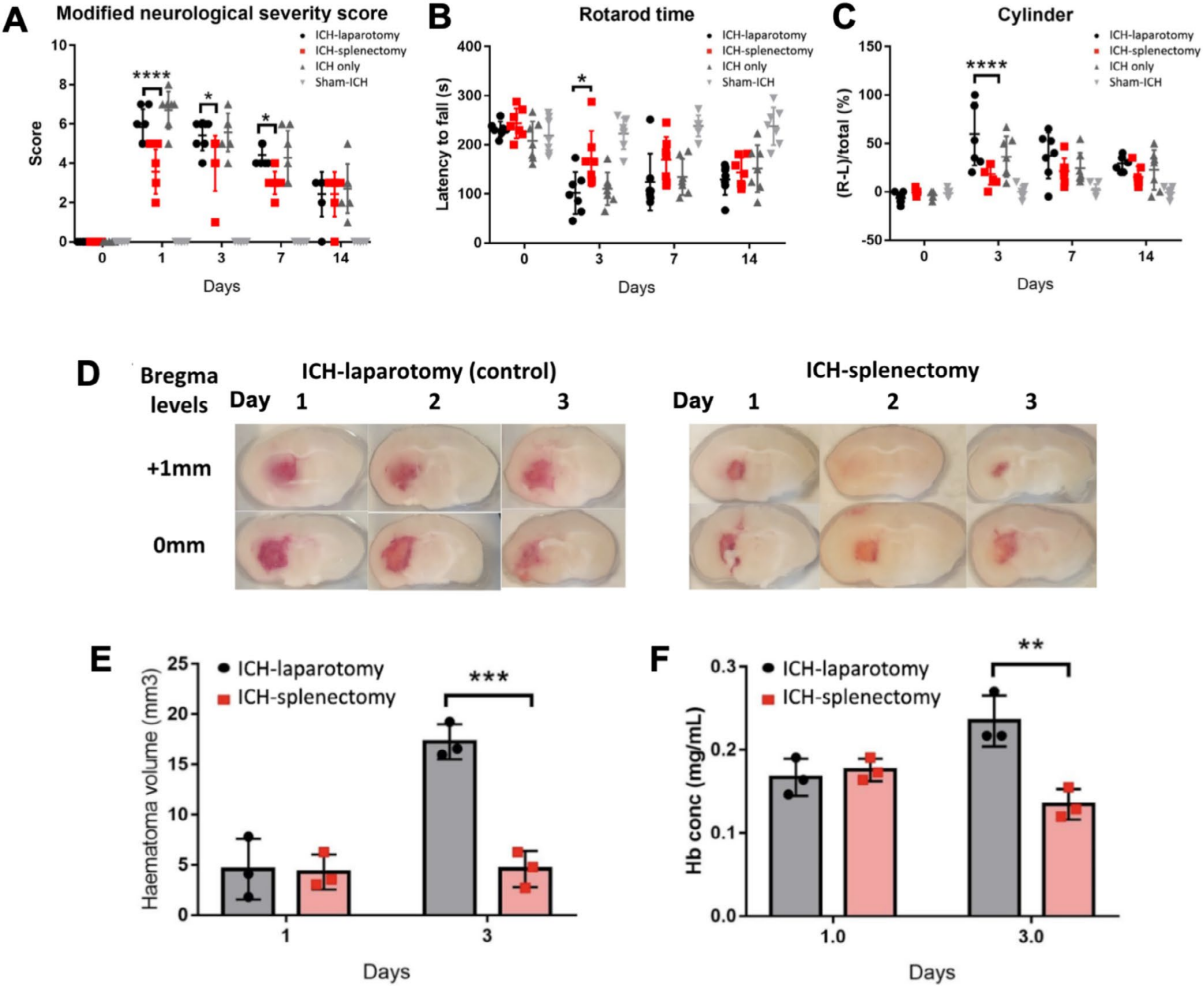
Splenectomy reduced haematoma size and improved neurological outcome. Mice were randomly divided into four groups: ICH‐splenectomy (ICH induction and laparotomy with splenectomy); ICH‐laparotomy (ICH induction and laparotomy without splenectomy); ICH‐only; and sham‐surgery only. (A) ICH‐splenectomy group had lower mNSS; (B) longer latency‐to‐fall time on rotarod test; and (C) lower forelimb asymmetry on cylinder test. The laterality index was calculated as: (ipsilateral forelimb usage count − contralateral forelimb usage count)/(total forelimb usage count). (D) Coronal brain slices of 1 mm thickness and (E) volumetric analysis, showing smaller haematomas in ICH‐splenectomy (4.59 ± 1.79 mm^3^ vs. 17.2 ± 1.73 mm^3^). (F) Hemoglobin assay showed lower hemoglobin (Hb) content (presented as Hb concentration) in splenectomised mice (0.126 ± 0.0222 mg/mL vs. 0.235 ± 0.0307 mg/mL). Data were presented as mean ± SEM, *n* = 3–7 mice per group; **p < 0.01; ***p < 0.001 by two‐way ANOVA.

The ICH‐splenectomy group scored significantly lower in mNSS on day 1 post‐ICH (****p < 0.0001), 3, and 7 (*p < 0.05) when compared to the ICH‐laparotomy group (Figure 1A). It also showed a significantly longer latency‐to‐fall time on the rotarod test (*p < 0.05) and more use of the impaired forepaw on the cylinder test (****p < 0.0001) on day 3 (Figure 1B,C).

Haematoma volumes in the ICH‐splenectomy and ICH‐laparotomy groups were comparable on day 1, but were significantly smaller in the ICH‐splenectomy group on day 3 (***p < 0.001). Haematomas in the ICH‐laparotomy group expanded between day 1 and day 3; no such changes occurred in the ICH‐splenectomy group (Figure 1D,E). The same trends were observed on hemoglobin assay, suggestive of delayed blood extravasation in the ICH‐laparotomy group, but not in the ICH‐splenectomy group. Hemoglobin content was lower on day 3 than on day 1 in the ICH‐splenectomy group, likely due to endogenous resorption (Figure 1F).

**FIGURE 2:**
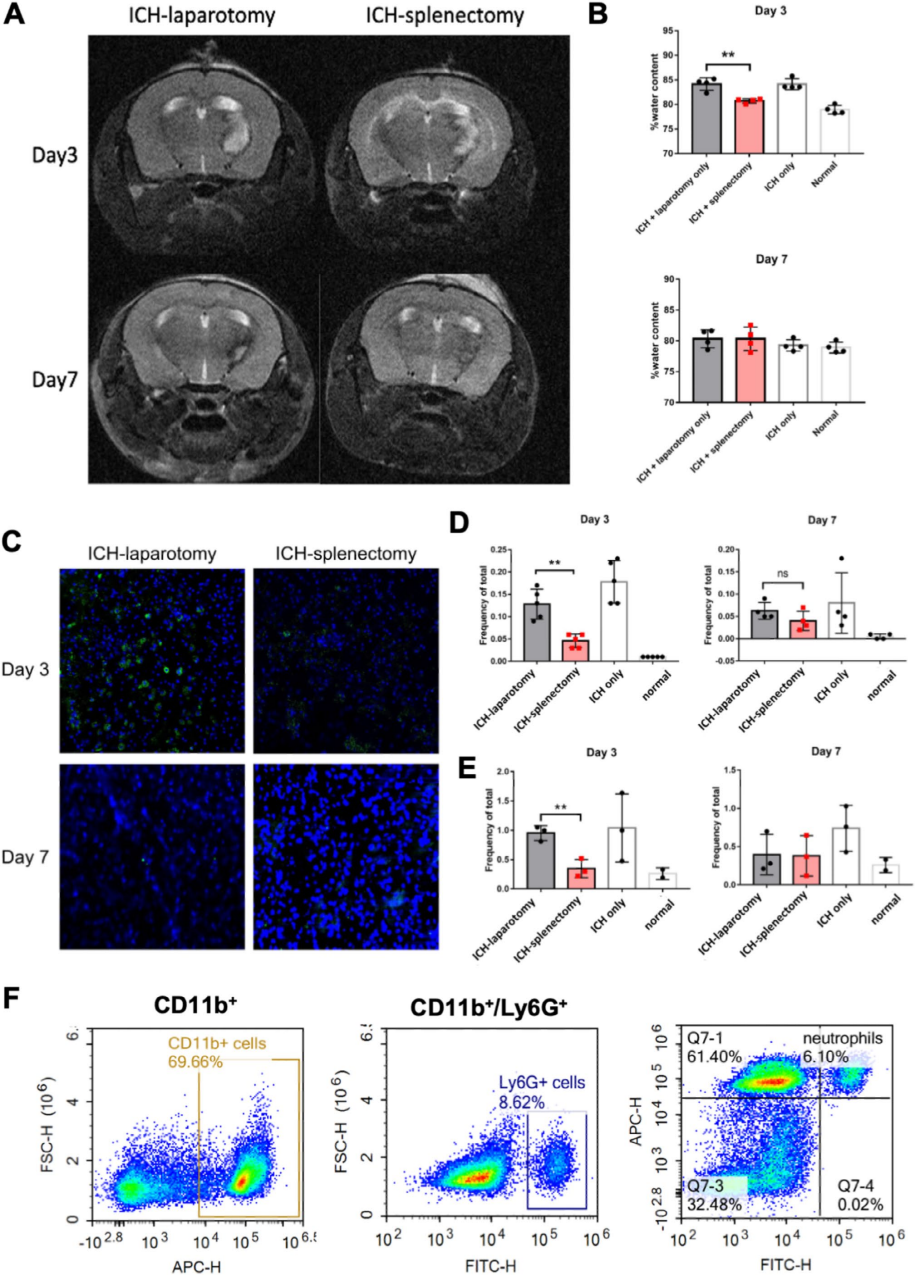
Splenectomy was associated with less cerebral oedema and reduced neutrophil infiltration to the brain. (A) Representative T2‐weighted MRI images. Oedema and haematoma size were observably smaller in the ICH‐splenectomy group. (B) Water content of the right hemisphere was significantly lower in the ICH‐splenectomy group on day 3 and day 7. Data are presented as mean ± SEM, *n* = 4 mice per group; **p < 0.01 by two‐way ANOVA. The brain's water content was calculated by: [(wet weight − dry weight)/wet weight] × 100%. (C) Representative images showing co‐localisation of neutrophil marker CD11b (green) with nuclei marker 4′,6‐diamidino‐2‐phenylindole (DAPI; blue) in peri‐haematoma regions. (D) Flow cytometry of brain samples, and (E) of peripheral blood samples, showing lower neutrophil counts in splenectomised mice, *n* = 3 mice per group; **p < 0.01 (F) Flow cytometry gating strategies using CD11b and Ly6G to identify neutrophils. The gating strategies were determined by fluorescence minus one (FMO) controls.

T2‐weighted MRI showed that each ICH mass lesion consisted of a hyperintense core (oxyhaemoglobin and extracellular methaemoglobin) and a hypointense rim (deoxyhemoglobin, methemoglobin, and hemosiderin). When compared with splenectomized mice, laparotomy‐only animals had observably bigger mass lesions (Figure 2A). Splenectomized mice had significantly higher water content within the affected cerebral hemisphere on day 3 (**p < 0.01), but not on day 7, indicating that a prior splenectomy resulted in less peri‐haematoma edema and less mass effect in the early stage of ICH (Figure 2B).

Immunofluorescent staining and flow cytometry showed that the ICH‐splenectomy group had a significantly lower intracerebral neutrophil count on day 3. Neutrophilic infiltration decreased by day 7, showing no between‐group differences (Figure 2C,D). The same was observed in peripheral blood samples (**p < 0.01) on day 3, reflecting changes in the brain (Figure 2E,F).

## 4 Discussion

Post‐ICH splenic contraction causes surges of circulating immune cells that contribute to inflammation, oedema, and tissue destruction in the brain [6, 7, 10, 11]. Our preliminary study, for the first time, demonstrated prior splenectomy would confer benefits after ICH, consistent with previous reports on ischaemic stroke [2, 3, 4, 12, 13, 14].

We found that splenectomised mice had lower neutrophil counts in the brain, which would have resulted in a less neuroinflammatory response and accounted for the less pronounced cerebral oedema seen in these animals. The absence of haematoma expansion suggests that their BBB was better preserved, in contrast with non‐splenectomised animals that showed marked extravasation. We surmise that the ensuing reduction in pressure effect and the amount of neurotoxic haemolytic substances within the peri‐haematoma region would, at least partially, account for the better functional outcomes in splenectomised mice [15, 16]. This finding is also consistent with a previous report demonstrating splenectomy attenuates ICH‐induced neurological impairment in mice [17].

The protective effects of splenectomy were short‐lived, however, being mostly detectable on day 3 but not beyond. This could be attributed to phenotypic changes of infiltrating immune cells, which tend to be pro‐inflammatory initially and anti‐inflammatory thereafter; a prior splenectomy would have been beneficial and counterproductive, respectively [18]. Furthermore, brain‐resident immune cells, such as microglia, would have existed in abundance throughout and eventually overcome the effects of splenectomy. Mechanistic studies focusing on different immune cell types and inflammatory cascades are required to assess the full impact of splenic response ablation.

Whether and how the effect‐window can be extended is another area for investigation. The lack of long‐term protection after splenectomy also happens in ischaemic stroke [19], where the timing of splenectomy and animal strains in regard to their differential autoimmune response can play important roles [20]. In humans, splenic response to stroke is affected by patient age and ethnicity, giving rise to additional considerations in clinical translation [6]. For example, the degree of splenic contraction on imaging studies and peripheral leucocyte count could serve as biomarkers in determining the utility of splenic ablation.

Granted that splenectomy is unlikely to be a viable treatment option clinically, our findings nonetheless provide rationale for exploiting other means of modulating the brain‐spleen crosstalk in the management of ICH [21, 22]. Pharmacological agents interfering with splenic activation or the release of inflammatory chemokines may hold promise [23, 24], while surgical interventions such as vagal nerve stimulation could inhibit splenic contraction after ICH [25], and partial splenic artery embolisation and hemodialysis could reduce the release of splenic immune cells and remove circulating chemokines, respectively [26]. Future studies should focus on the optimal mode and timing of intervention, taking into consideration the characteristics of the innate immune response and potential side effects of systemic immuno‐depletion.

## Author Contributions

C.H.L. and H.T.S. performed research and wrote the manuscript; J.L. and K.M.K. contributed to the development of methodology and interpretation of results; G.K.‐K.L. provided the study materials and supervised the entire study.

## Conflicts of Interest

The authors declare no conflicts of interest.

## Data Availability

Data sharing not applicable to this article as no datasets were generated or analyzed during the current study.
